# Whole-Body Cryostimulation in Functional Neurological Disorders: A Case Report

**DOI:** 10.3390/healthcare12010071

**Published:** 2023-12-28

**Authors:** Federica Verme, Jacopo Maria Fontana, Paolo Piterà, Angelo Alito, Silvia Saffioti, Gabriele Baccalaro, Giuliano Zebellin, Paolo Capodaglio

**Affiliations:** 1 Research Laboratory in Biomechanics, Rehabilitation and Ergonomics, Istituto Auxologico Italiano, IRCCS, San Giuseppe Hospital, Piancavallo, 28824 Verbania, Italy; j.fontana@auxologico.it (J.M.F.); p.pitera@auxologico.it (P.P.); silviasaffioti7@gmail.com (S.S.); g.baccalaro@auxologico.it (G.B.); g.zebellin@auxologico.it (G.Z.); p.capodaglio@auxologico.it (P.C.); 2 Department of Biomedical, Dental Sciences and Morphological and Functional Images, University of Messina, 98125 Messina, Italy; alitoa@unime.it; 3 Department of Surgical Sciences, Physical Medicine and Rehabilitation, University of Torino, 10126 Torino, Italy

**Keywords:** chronic pain, conversion disorder, functional neurological disorders, rehabilitation, whole-body cryostimulation

## Abstract

Functional neurological disorders (FNDs) are complex disabling conditions requiring a multiple rehabilitation intervention. Here, we propose a new use of whole-body cryostimulation (WBC) that was implemented in a multidisciplinary rehabilitation programme in a wheelchair-ridden woman diagnosed with FND and other comorbidities. WBC is a promising adjuvant treatment in various conditions of rehabilitation interest, mainly because of its wide range of rapid effects, from anti-inflammatory to pain and autonomic modulating effects. The 4-week program included physiotherapy, nutritional intervention, psychological support, and WBC (−110 °C for 2 min). Questionnaires to assess disease impact, pain level, fatigue and sleep quality were administered. At discharge, improvements in body composition, haematological biomarkers, physical performance, and questionnaire scores were observed. The patient was able to walk independently with a walker for medium distances and reported unprecedented improvements, particularly in functional parameters and questionnaire scores. Although the extent to which WBC per se contributed to the measured improvements cannot be ascertained, subjective reports and our clinical observations indicate that WBC, the only intervention not previously experienced by the patient, acted as a booster for the rehabilitation interventions. Further research will be necessary to rule out any possible placebo effect and to confirm the effects of WBC on FND.

## 1. Introduction

Functional neurological disorders (FND) (also called conversion disorders or functional neurological symptom disorders) are relatively common problems in neurologic practice. The diagnosis is based on findings in the neurological examination demonstrating that the symptom lasting for at least 6 months is incompatible with a structural neurological illness [1]. In FND, the cardinal symptom is limited solely to neurological symptoms: alterations in voluntary motor, cognitive, or sensory function that are not compatible with any recognized neurological condition. According to the DSM-5 criteria, the clinician’s diagnosis is made on the basis of the positive findings of the examination demonstrating preserved neurological function [2]. Disability in FND is similar to that in structural disease, and neurological outcome at 8 months is poor, with over 50% of patients showing no improvement [1]. Among therapeutic options, multidisciplinary rehabilitation is an important component of symptomatic and supportive treatment for FND [3]. Participation in a treatment process that changes “the way the brain processes information” is essential to minimise the tendency to express distress through physical symptoms. Physiotherapy and physical therapies are first-line treatments for patients with motor FND and have the goal of changing the processing of complex motor programs and facilitating engagement in more adaptive patterns of movement. There is evidence that they are helpful in treating the motor manifestations of FND [4,5]. Clinical judgment should be used when it is appropriate to initiate medications and psychotherapy to treat comorbid psychiatric conditions. Cognitive behavioural therapy has been shown to be beneficial in treating FND, and a variety of other psychotherapies have been proposed [3,6]. Patients with FND require an integrated multidisciplinary approach and both the intensity and educational component of the program appear to be important factors for success.

Whole-body cryostimulation (WBC) is a physical treatment where the entire body is exposed to cryogenic temperatures (−110 °C to −140 °C) for 2–3 min. Exposure to these temperatures is known to reduce pain and inflammation and improve metabolic parameters (thermogenesis, lipid profile, insulin sensitivity, and glucose utilisation), as well as depression, anxiety, and sleep quality [7,8,9,10,11]. The analgesic effect of WBC originates from the sudden drop in skin temperatures and the activation of the sympathetic system, which modulates the pain response by slowing down nerve conduction velocity in pain fibres (i.e., C-fibres), modulating neurotransmitters involved in pain signalling, inhibiting sensory receptors and their connections to proprioceptors, and by releasing analgesic factors such as serum beta-endorphin and norepinephrine. Together with the latter, the increase in parasympathetic tone results in reduced fatigue, muscle tension and soreness, improved mood, and symptoms of depression, ultimately leading to a reduction in pain perception [12]. For all these reasons, cycles of WBC have led to improved rehabilitation outcomes in patients with conditions such as multiple sclerosis [13], post-COVID-19 condition [14], rheumatoid arthritis [15], ankylosing spondylitis [16], polymyalgia rheumatica [17], and fibromyalgia [18,19,20,21]. Despite its 40-year-long use worldwide, WBC is associated with rare and mostly transient adverse effects. The safety of WBC treatment and possible adverse events have recently been reviewed [22]. WBC appears a safe and well-tolerated treatment when accurate medical screening is performed.

To the best of our knowledge, no studies have so far investigated the effects of WBC on FND. Given the above-mentioned wide spectrum of rapid effects ascribed to WBC, the reported good tolerance to this treatment, the absence of clinical contraindications, and the involvement of the patient in an engaging and intense physical treatment experience, which could potentially contribute to psychophysical well-being, particularly in functional conditions, we proposed WBC for symptoms management as part of a multidisciplinary rehabilitation program.

## 2. Detailed Case Description

T.C., a 61-year-old woman, wheelchair-ridden for the last year, was admitted to the Rehabilitation Unit of San Giuseppe Hospital, Istituto Auxologico Italiano, Piancavallo (VB), Italy for a multidisciplinary program with the following diagnoses: FND, polyarthralgia (particularly lumbar spine with disc protrusions, and knee arthritis), widespread myalgia, sleep disorder, morbid obesity, arterial hypertension, urinary incontinence, gastroesophageal reflux.

Medical history. After the age of 18, the patient progressively gained body weight. In 2013, she experienced distress due to an abrupt dismissal from work. Thereafter, physical activity levels dropped dramatically and eating behaviour became dysfunctional. She was diagnosed with dysthymia and reactive anxiety for which psychological and pharmacological support (benzodiazepines and selective serotonin reuptake inhibitors) were prescribed, with no subjective benefit.

In 2017, her brother, to whom she was very close, diagnosed with Amyotrophic Lateral Sclerosis, passed away.

In the following year, she was diagnosed with an anxiety disorder causing atypical chest pain, sleep disturbances, and distal motor deficit of the upper limbs (EMG and EEG were negative) and given a medical prescription of antidepressants and pregabalin with psychological support. No evidence of autoimmune or systemic inflammatory disease was found, and an MRI of the lumbar spine documented discopathy with left L2–L3 intraforaminal protrusions, narrowing of the spinal canal at L3–L4 and L4–L5, and thickening of the yellow ligaments. Bone scintigraphy revealed polyarticular oedema, juxta-articular osteoporosis, and enthesopathy of the Achilles tendon and plantar fasciae. A diagnosis of fibromyalgia was formulated. In April 2021, she was showing difficulty in walking, “rigidity” of the lower limbs, and widespread pain. The EMG findings were normal. MRI of the brain revealed a Dandy–Walker malformation. Neuropsychological tests revealed mild memory and praxic impairments in both the visual-spatial and episodic anterograde components, with a normal Mini-Mental State Examination score. The diagnosis of fibromyalgia was confirmed.

In May 2021, she reported a motor deficit in the left side of the body, left facial clonus, dysarthria, and oral rhyme deviation, but a CT scan was negative. A diagnosis of FND was formulated, and pharmacological therapy (duloxetine, topiramate, benzodiazepines) was prescribed. In January 2022, stress urinary incontinence with detrusor hyperactivity and low compliance bladder with interrupted flow was diagnosed. In June 2022, EEG showed global minimal slowing of the electric cerebral activity with an excess of slow intermittent activity in the frontotemporal site. Walking was only possible for a few steps with double support and steppage gait. Obesity was also present. The patient had been wheelchair-bound from that time. In January 2023, a difficulty in sit-to-stand was evident. She unsuccessfully underwent outpatient physical therapy and weight management interventions. In July 2023, signs of cortical release (palmomental and muzzle-glabellar reflexes) required a PET/CT scan to rule out possible frontotemporal dementia, but no abnormalities were found. Cognitive functions were normal, except for a slight reduction in working and short-term memory. She needed assistance with dressing/undressing and personal hygiene.

Clinical Examination. On admission to our Rehabilitation Unit, she presented with morbid obesity (body weight 113.2 kg, height 167 cm, BMI: 40.5) and lipo-lymphedema in her lower limbs. She was complaining of diffuse pain and weakness, and her fibromyalgia trigger points were positive. From a functional point of view, sit-to-stand was performed slowly and only with direct assistance, she was able to move a few steps with double support and assistance, and bilateral steppage and dyspnoea were evident. Her upper limb muscular strength (Mingazzini I manoeuvre) was bilaterally reduced, but when verbally encouraged she was able to hold the position for 15 s. Muscular strength in the lower limbs (Mingazzini II manoeuvre) was reduced, but, if verbally encouraged, she was able to hold the position for 10 s. Segmentary strength was difficult to assess because of the lack of voluntary recruitment of muscles on demand, whereas, in the functional context, specific muscles were activated. Other central or peripheral neurological signs were absent. Spinal digital compression was painful, and the articular range of motion in the lower limbs was limited because of adiposity. Overall, the clinical picture showed functional limitation secondary to multifactorial aetiology in chronic degenerative pain, a psychiatric component, fibromyalgia syndrome, and morbid obesity.

Rehabilitation interventions. The following interventions were planned progressively during a 4-week hospital stay: mobilisation (first passive, then assisted, and finally active) of the 4 limbs and girdles, low-intensity arm ergometer exercises to increase aerobic capacity and exercise tolerance, gradual isometric and isotonic muscle strengthening aimed at functioning in daily life, drainage techniques and pressure therapy for lower limb lymphedema, proprioceptive exercises, training of postural transfers, trunk balance, sit-to-stand, aids-assisted standing, and specific gait training with progressive walking aids. Physical therapies (TENS at the spinal level, and diathermy therapy at the knee level) were also provided. Psychiatric/psychological support was provided for education and awareness of possible correlations among depressive symptoms, chronic pain, obesity and functional syndromes along with behavioural therapy for pain management, monitoring of mood, sleep, pain, and eating behaviour with caregivers’ involvement.

Nutritional intervention. A 1300 kcal diet was divided into three meals and composed as follows: 70 g proteins (21%), 42 g lipids (29%), and 162 g carbohydrates (50%).

WBC. The patient underwent a total of nine daily WBC sessions in a cryo-chamber (Artic, CryoScience, Rome, Italy) where she was exposed to extremely cold and dry air at −110 °C for two minutes. Before starting the treatment, the patient underwent a medical examination to rule out any contraindications according to Bad Voslau’s guidelines [23]. She was asked to remove glasses and metal accessories and to wear light clothing, such as a T-shirt, running shorts, an earmuff band, gloves, socks (pulled up to the knee), and rubber slippers. The patient was encouraged to breathe calmly throughout the session, which was under the supervision of trained personnel.

Outcome measures. Anthropometrics and body composition (body weight, body mass index (BMI), fat mass (FM) and fat-free mass (FFM)), haematological biomarkers, and physical performance tests were measured before and after the completion of the rehabilitation program. We performed the Functional Independence Measure (FIM) [24], the Timed Up and Go test (TUG) and the Visual Analogue Scale (VAS) for pain and disability. The patient was also asked to fill in several validated questionnaires: the Revised Fibromyalgia Impact Questionnaire (FIQR) [25], the Fatigue Severity Scale (FSS) [26], a numeric rating scale for pain (NPRS) [27], the Central Sensitization Inventory (CSI) [28], the Brief Pain Inventory (BPI) [29], the Depression Anxiety Stress Scales-21 (DASS-21) [30], the World Health Organisation—Five Well-Being Index (WHO-5) [31], and the Pittsburgh Sleep Quality Index (PSQI) [32].

Results. After the comprehensive rehabilitation program, the patient achieved adequate weight loss, improved trunk mobility, and was able to walk medium distances with a four-wheel walker, even outdoors. All indicators considered improved (see Table 1), and the questionnaire scores showed a clear improvement in the patient’s disease impact, fatigue, pain, general state of well-being, and sleep quality (see Table 2).

## 3. Discussion

FNDs are complex psychological disorders which call for a multidisciplinary approach to manage the multiple symptoms and clinical presentations. Psychological interventions have traditionally been considered the core treatment, but a major role for rehabilitation interventions and physical therapies is nowadays acknowledged when motor symptoms prevail [33]. The nature of the condition and its poor recovery prognosis pose a challenge to clinicians, especially in the selection of the most appropriate treatment, which should address both physical and psychological aspects. FNDs can be associated with several comorbidities, including chronic pain, fatigue, depression, and anxiety. When these conditions are present, patients may benefit also from specific interventions [33].

We have proposed the application of WBC within a multidisciplinary rehabilitation program in a wheelchair-ridden woman diagnosed with FND, based on the broad spectrum of positive effects attributed to WBC, its known rapid analgesic/anti-inflammatory effects and the nature of the treatment, which engages the patient in an intense physical experience with the potential to address also the psychological components of the clinical picture. Involvement in a comprehensive programme can increase active patients’ participation and improve adherence to treatment. Our rehabilitation programme led to the improvement of the patient’s motor, psychological, and anthropometric parameters, to the point that at the end of her hospital stay, she was able to walk independently with a walker for medium distances, even outdoors.

An “off-label” use of WBC in different clinical conditions is not novel. Recent studies by our group have demonstrated the positive effects of WBC on the post-COVID-19 condition and polymyalgia rheumatica [14,17,34]. These benefits can be attributed to the known effects of WBC on reducing pain, fatigue, and inflammation, and improving mood and sleep quality [7,8,15,19,35].

In Poland, WBC has become a standard treatment in neurological and musculoskeletal disorders, and preliminary evidence of WBC benefits in mental disorders has been reported [36]. In a case–control study by Miller et al., WBC sessions induced a significant improvement in the functional status and in the feeling of fatigue in patients with multiple sclerosis (MS) [13]. Rymaszewska’s group investigated the benefits of WBC on several conditions. The results of a 2008 study suggested a possible role for WBC as a short-term adjuvant treatment for mood and anxiety disorders [7]. In a study on MS patients, WBC in addition to physical exercise training proved to be effective in improving psychophysical well-being and reducing anxiety and depressive symptoms [37]. The results of another study showed that WBC reduces mental health deterioration, especially in mood disorders (such as depression), and can improve well-being and quality of life [38]. In a recent randomised controlled trial that analysed the effects of WBC on cognitive function in patients with mild cognitive impairment, a significant improvement in general cognitive function and episodic memory was shown in the group undergoing WBC [36].

Some limitations in this first report must be taken into account. The patient’s positive emotional involvement in the treatment and the high degree of satisfaction may have played a role in the observed global functional improvement, so a placebo effect cannot be ruled out. Above all, we cannot ascertain to what extent WBC per se may have contributed to the outcomes, as the patient underwent a comprehensive rehabilitation program including nutrition, physiotherapy, and psychological support in addition to WBC.

However, the patient’s subjective perception was that, from the very early sessions, WBC provided a rapid feeling of well-being and reduced the perception of fatigue and pain. Tests showed unprecedented improvements, particularly in physical performance, to the point where she no longer needed a wheelchair for mobility, as well as in symptoms related to pain, fatigue, sleep quality, and well-being. Our clinical hypothesis is that WBC, a treatment never before experienced by the patient, may have acted as a booster for the rehabilitation intervention. Further research on a larger sample will be needed to confirm the effects of WBC on FND.

## Figures and Tables

**Table 1:** Pre-post total scores and percentage change in anthropometric measurements (body weight, BMI, FM, and FFM) and functional indicators (FIM, TUG, VAS pain lower limbs and lumbar spine, VAS disability, and trunk mobility). The percentage change (∆%) was calculated using the formula: ∆% = (POST − PRE)/PRE × 100.

**Anthropometric Measurements**				
		**PRE**	**POST**	**∆%**
Body weight (kg)		113.2	107	−5.48
BMI (kg/m^2^)		40.5	38.3	−5.43
FM (%)		56	54.1	−3.39
FFM (%)		43.7	45.9	5.03
**Functional indicators**				
		**PRE**	**POST**	**∆%**
FIM		96	109	13.54
TUG (s)		38.05	20.22	−46.86
VAS	Pain (Lower limbs)	80	40	−50.00
	Pain (Lumbar spine)	90	50	−44.44
	Disability	100	70	−30.00
Trunk	Bending (°)	40	70	75.00
	Extension (°)	5	10	100.00
	Left Inclination (°)	10	15	50
	Right Inclination (°)	10	15	50

BMI, body mass index; FIM, Functional Independence Measure; FM, fat mass; FFM, fat-free mass; TUG, Timed Up and Go test; VAS, visual analogue scale.

**Table 2:** Pre-post total scores and percentage change in Questionnaire (FIQ, FSS, NPRS, CSI, BPI, DASS-21, WHO-5 and PSQI) scores. The percentage change (∆%) was calculated using the formula: ∆% = (POST − PRE)/PRE × 100.

Questionnaires (Total Scores)				
		PRE	POST	∆%
FIQR		71.83	26.33	−63.34
FSS		33	15	−54.54
NPRS		9	1	−88.89
CSI		42	23	−45.24
BPI	Pain Severity	5.75	1.75	−69.56
	Pain Interference	5.43	2.29	−57.83
DASS-21	Depression	10	6	−40
	Anxiety	10	4	−60
	Stress	12	6	−50
WHO-5		72	88	22.22
PSQI		12	8	−33.33

BPI, Brief Pain Inventory; CSI, Central Sensitization Inventory; DASS-21, Depression Anxiety Stress Scales-21; FIQR, Revised Fibromyalgia Impact Questionnaire; FSS, Fatigue Severity Scale; NPRS, Numeric Pain Rating Scale; PSQI, Pittsburgh Sleep Quality Index; WHO-5 World Health Organisation—Five Well-Being Index.

## Data Availability

The data presented in this study are available within the article.
